# Low rates of structured advance care planning documentation in electronic health records: results of a single-center observational study

**DOI:** 10.1186/s12904-022-01099-9

**Published:** 2022-11-22

**Authors:** Adela Wu, Robert J. Huang, Gabriela Ruiz Colón, Chris Zembrzuski, Chirag B. Patel

**Affiliations:** 1 Department of Neurosurgery, Stanford Health Care, 300 Pasteur Drive, Palo Alto, CA 94304 USA; 2 Division of Gastroenterology, Department of Medicine, Stanford University School of Medicine, Palo Alto, CA 94304 USA; 3 Stanford University School of Medicine, Palo Alto, CA 94304 USA; 4 Rowan School of Osteopathic Medicine, Stratford, NJ USA; 5 Department of Neuro-Oncology, The University of Texas MD Anderson Cancer Center, 1515 Holcombe Blvd, Unit 1002, BSRB S5.8116B, Houston, TX 77030 USA

**Keywords:** Advance care planning, Outpatient, Ambulatory, Clinic, Documentation, Electronic health record

## Abstract

**Background:**

Proper advance care planning (ACP) documentation both improves patient care and is increasingly seen as a marker of high quality by governmental payers. The transition of most medical documentation to electronic health records (EHR) allows for ACP documents to be rapidly disseminated across diverse ambulatory practice settings. At the same time, the complexity and heterogeneity of the EHR, as well as the multiple potential storage locations for documentation, may lead to confusion and inaccessibility. There has been movement to promote structured ACP (S-ACP) documentation within the EHR.

**Methods:**

We performed a retrospective cohort study at a single, large university medical center in California to analyze rates of S-ACP documentation. S-ACP was defined as ACP documentation contained in standardized locations, auditable, and not in free-text format. The analytic cohort composed of all patients 65 and older with at least one ambulatory encounter at Stanford Health Care between 2012 and 2020, and without concurrent hospice care. We then analyzed clinic-level, provider-level, insurance, and temporal factors associated with S-ACP documentation rate.

**Results:**

Of 187,316 unique outpatient encounters between 2012 and 2020, only 7,902 (4.2%) contained S-ACP documentation in the EHR. The most common methods of S-ACP documentation were through problem list diagnoses (3,802; 40.3%) and scanned documents (3,791; 40.0%). At the clinic level, marked variability in S-ACP documentation was observed, with Senior Care (46.6%) and Palliative Care (25.0%) demonstrating highest rates. There was a temporal trend toward increased S-ACP documentation rate (*p* < 0.001).

**Conclusion:**

This retrospective, single-center study reveals a low rate of S-ACP documentation irrespective of clinic and specialty. While S-ACP documentation rate should not be construed as a proxy for ACP documentation rate, it nonetheless serves as an important quality metric which may be reported to payers. This study highlights the need to both centralize and standardize reporting of ACP documentation in complex EHR systems.

**Supplementary Information:**

The online version contains supplementary material available at 10.1186/s12904-022-01099-9.

## Background

Advance care planning (ACP) allows patients to make informed decisions about their medical care. Studies have shown that ACP improves communication between patients and providers, reduces unnecessary hospitalizations, and enhances patients’ quality of life [1, 2]. ACP is an ongoing process that involves the identification of values important to the patient as well as preferences regarding medical treatment, which would be documented in the medical record along with a surrogate decision maker if applicable [3]. Therefore, successful ACP requires accurate and timely documentation of goals of care discussions.

The transition of most medical documentation to electronic health records (EHR) allows for ACP documents to be rapidly disseminated across diverse ambulatory practice settings. However, the majority of ACP in the EHR is documented in the form of narrative free text, data which is difficult to locate and access. For providers to understand and act on a patient’s wishes, they require documentation that is accessible, standardized, and up-to-date. There has been movement toward structured-ACP (S-ACP) documentation [4–6]. S-ACP refers to ACP which is readily accessible, structured, auditable, and contained in standardized locations. Lakin et al. describes S-ACP as EHR data elements, such as advance directives and out-of-hospital code statuses, which encompass unique ACP documentation and information [4]. In contrast to patient preferences buried within free-text, such as progress notes, S-ACP has the potential to improve adherence to discussion and documentation, particularly within the EHR [7]. Moreover, S-ACP is increasingly being adopted as a key quality metric by payers [8]. The Center for Medicare and Medicaid Services (CMS) has adopted an ACP measure to ensure the existence of these critical patient-provider discussions in claims.

While prior studies have evaluated rates of ACP, few have analyzed specifically rates of S-ACP [9, 10]. Thus, we evaluated de-identified data derived from outpatient clinics at a single institution and examined rates of S-ACP documentation at the clinic level. We also sought to understand clinic- and provider-level factors which are related to S-ACP completion.

## Methods

### Ethics and approval

The Stanford University Institutional Review Board waived review of this study based on its classification as a quality improvement initiative.

### Data sources

The study was based at Stanford Health Care (SHC), a vertically-integrated healthcare system comprising three hospitals and multiple clinics and health centers located throughout Northern California. SHC utilizes an EHR system (Epic Systems, Verona, WI) for all outpatient and inpatient clinical encounters.

### Data capture and cohort creation

As part of an ongoing quality initiative, the SHC Privacy Office generated a report (accessed by AW and CBP) which incorporated data from January 2012 to May 2020. The purpose of this report was to aggregate statistics on ACP reporting and documentation, with the goal of improving reporting rates for commercial and governmental payers. This report captured data from all ambulatory clinics, all clinical departments, and all individual providers at SHC.

The primary metric was availability of S-ACP documentation in the EHR. S-ACP refers to ACP which is readily accessible, in a structured format and standardized location within the EHR, and is easily auditable through automated mechanisms [6]. S-ACP was captured through the following six sources: ACP ‘Smartform’ documentation designed by SHC through its EHR, existence of Current Procedural Terminology (CPT) coding, existence of appropriate diagnosis code in either the “Problem List” or “Encounter Diagnoses”, existence of a specified document type scanned in the media tab (Physician Orders for Life Sustaining Treatment [POLST], Advanced Directive, Living Will, or Do Not Resuscitate [DNR] directive), or the listing of an active surrogate decision maker in the EHR. A comprehensive list of definitions for S-ACP is provided in Supplemental Table 1. Existence of any of these six items in the 12 months prior to the reporting period was considered documentation of S-ACP.

**Table 1:** S-ACP documentation rate by clinic: encounter-level structured-advanced care planning (S-ACP) documentation rate by ambulatory clinic. Analysis restricted to clinics with at least 500 eligible encounters

**Clinic name**	Denominator	Numerator	Percentage
Audiology	920	100	10.9
Blood and bone marrow	1,620	187	11.5
Breast oncology	2,888	179	6.2
Cancer genetics	1,226	95	7.8
Cardiology	25,807	1,896	7.4
Cardiovascular surgery	4,227	206	4.9
Cancer Center interventional radiology	1,733	164	9.5
Cancer Center reconstructive	827	37	4.5
Chest Clinic	9,019	841	9.3
Cutaneous oncology	3,217	315	9.8
Dermatology	26,629	2,321	8.7
Digestive Health Center	13,200	955	7.2
Endocrinology	8,168	727	8.9
Endocrine oncology	511	40	7.8
Express Care	9,896	1,561	15.8
Eye Institute	28,756	2,153	7.5
Family medicine	3,230	434	13.4
GI surgery	530	41	7.7
GI oncology	6,962	470	6.8
Gynecology	5,010	409	8.2
Gyn-oncology	1,854	110	5.9
Head neck oncology	4,222	300	7.1
Hematology	5,021	604	12.0
Immunology	3,789	247	6.5
Infectious disease	5,027	505	10.1
Internal medicine	13,888	1,605	11.6
Integrative medicine	975	127	13.0
Kidney	5,323	489	9.2
Liver	4,844	319	6.6
Lymphoma	1,934	118	6.1
Neurooncology	2,205	180	8.2
Neurology	23,110	1,656	7.2
Neurosurgery	14,480	819	5.7
Orthopedics	40,643	2,423	5.9
Otolaryngology	19,179	1,222	6.4
Pain management	7,556	492	6.5
Palliative medicine	1,295	324	25.0
Plastic surgery	3,453	259	7.5
Psychiatry	3,274	378	11.6
Preanesthesia	41,386	2,324	5.6
Radiation oncology	6,453	560	8.7
Sarcoma	650	39	6.0
Sleep	8,297	666	8.0
Social work	701	106	15.1
Senior care	2,629	1,225	46.6
Primary care	5,099	657	12.9
Transplant outreach	1,702	19	1.2
Thoracic oncology	3,642	300	8.2
Vascular	9,606	764	7.9
Urology	11,450	1,029	9.0
Urologic oncology	7,715	485	6.3

All unique patients 65 years and older with at least one clinical encounter at an SHC outpatient clinic over the study period were included for analysis. The report included only patients in this age range due to the CMS Innovation’s Bundled Payments for Care Improvement Advanced Model, which rewards healthcare providers who engage in provision of high-quality care, including ACP documentation [8]. Any patient with CPT code G9692 denoting hospice use during any point of the reporting period was excluded from analysis due to the parameters of the reporting mechanism. As patients often had multiple encounters, the number of encounters (*N* = 1,350,787) exceeded the number of unique patients (*N* = 187,690).

### Endpoints and analyses

The rate of S-ACP documentation in a reporting period was defined as the proportion of all unique individuals who met inclusion criteria (denominator) with S-ACP documentation in the EHR (numerator). Rates of S-ACP documentation were compared by month over the study period. Rates of S-ACP documentation were also compared for clinics, provider departments, and payers. We restricted analysis to clinics with 500 eligible encounters over the study period. Clinics were additionally classified as either ‘cancer’ or ‘non-cancer’ based on patient population served and their affiliation with the Stanford Cancer Center. Providers were classified based on training and clinical role (*e.g.* physician, psychologist, nurse practitioner), and we restricted analysis to provider types that logged more than 450 encounters over the study period.

The χ2 test and analysis of variance were used to assess differences in S-ACP documentation rate between and among clinics, provider departments, and payer. The Cochran-Armitage Test was used to assess linear trends in S-ACP documentation rate over time. All analyses were performed using R software (version 3.5).

## Results

### Rates and methods of S-ACP documentation

**Fig. 1:**
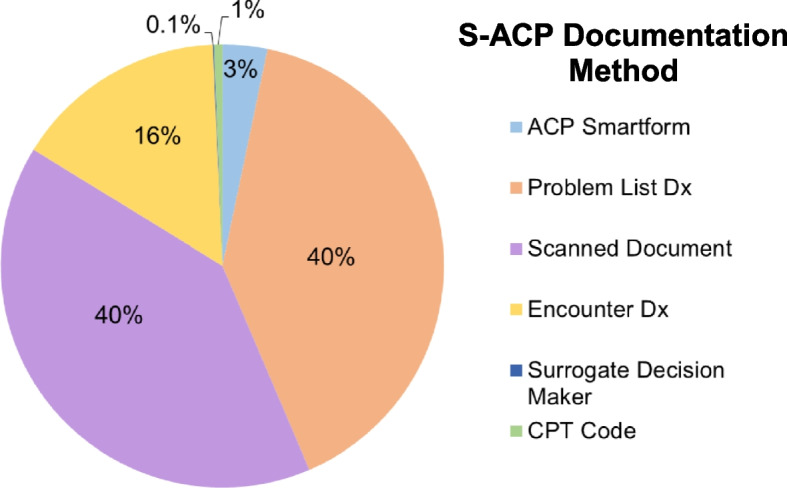
Title: Location of structured advance care planning (S-ACP) documentation in the electronic health record (EHR). Abbreviations: current procedural terminology (CPT), diagnosis (Dx)

The cohort comprised of 187,690 unique patients and 1,350,787 encounters over the study period. Of 187,690 patients, 7,902 (4.2%) had completed at least one form of S-ACP documentation. These 7,902 individuals had recorded 9,431 unique S-ACP documentation instances (with some individuals having multiple forms of S-ACP documentation) in the EHR. The most common methods of documentation were scanned documents (3,791; 40.0%) and by problem list diagnoses (3,802; 40.3%) (Fig. 1). Documentation of surrogate decision maker was the least common form of S-ACP documentation (0.1%).

**Fig. 2:**
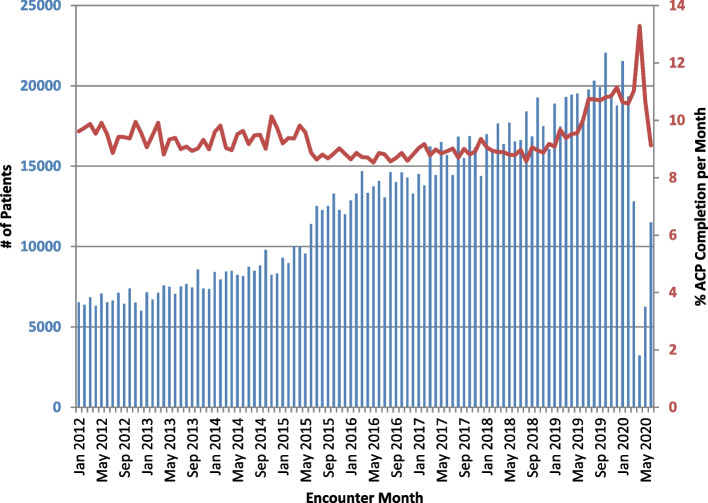
Encounter-level S-ACP documentation rate by month. Number of ambulatory clinic encounters per month from January 2012 to May 2020 is denoted by blue bars (left y-axis). Proportion of encounters with structured advanced care planning (S-ACP) documentation per month denoted by the red line (right y-axis). S-ACP documentation rate increased over the study period (*p *< 0.001)

Monthly S-ACP documentation rates across the entire healthcare system were calculated across the 101-month reporting period (January 2012-May 2020). In Fig. 2, the proportion of encounters in each study month with S-ACP documentation is depicted. Notably, as some patients have multiple encounters, the encounter-level S-ACP documentation rate differs from the patient-level S-ACP documentation rate. The median number of encounters during the study period was 12,942 per month, and the median encounter-level S-ACP documentation rate was 9.1% per month. There was a statistically significant increase in monthly S-ACP documentation rate between January 2012 and May 2020 (*p *< 0.001).

### Analysis by clinic and provider

**Fig. 3:**
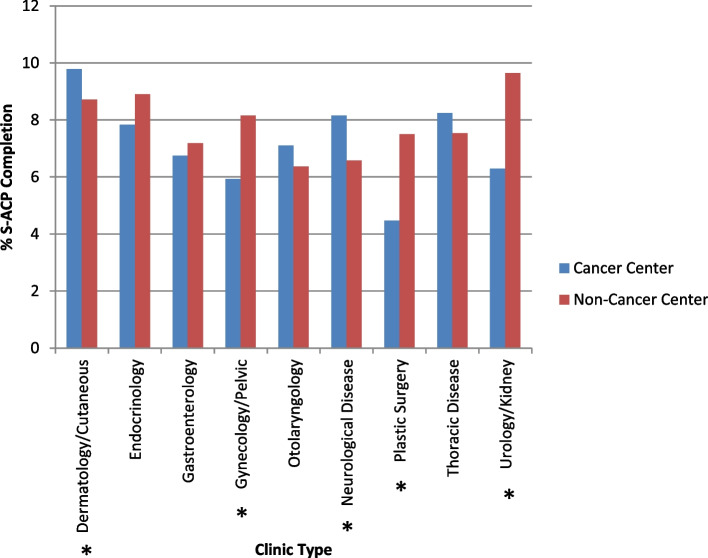
S-ACP documentation rates by clinic, stratified by oncologic status: Clinics which primarily served patients with oncologic diagnoses were classified as cancer, whereas clinics which primarily served patients with non-oncologic diagnoses were classified as non-cancer. * denotes significance at *p* < 0.05. Abbreviations: structured advance care planning (S-ACP)

The S-ACP completion rate among clinics with at least 500 eligible encounters ranged from 1.2% to 46.6% (Table 1). There existed significant differences in S-ACP documentation rate among clinics (*p* < 0.001). The clinics with the highest S-ACP documentation rates were Senior Care (46.6%), Palliative Medicine (25.0%), Express Care (15.8%), Social Work (15.1%), and Family Medicine (13.4%). Within medical specialties, clinics were further compared based on their affiliation with the Cancer Center (Fig. 3). While we expected Cancer Center clinics to have higher S-ACP documentation rates, this was not observed in the data; in certain cases, Cancer Center clinics even had significantly lower rates of S-ACP documentation compared to non-Cancer Center clinics (Gynecology/Pelvic: *p* = 0.002; Plastic Surgery: *p* = 0.002; Urology/Kidney: *p* < 0.0001).

Encounter-level S-ACP documentation was compared for providers (*e.g.* physician, psychologist, case manager) (Supplemental Table 2). Among provider classes, clinics run by Social Workers demonstrated the highest S-ACP documentation rate (28.9%) followed by Clinical Nurse Specialists (19.5%). Resident physician-directed clinics (11.5%) demonstrated higher S-ACP documentation compared to attending physician-directed clinics (9.3%, *p* < 0.001). Also notably, Nurse Practitioners (9.8%) and Physician Assistants (9.7%) demonstrated comparable if not better S-ACP documentation compared to Attending Physicians (9.3%).

## Discussion

S-ACP documentation is a recent though increasingly-used metric of high-quality care that is audited by payers and health system administrators. As the majority of healthcare providers now practice in systems served by EHRs, S-ACP provides important advantages to free-text ACP documentation, including standardization, ease-of-access, lower provider-level variability, and auditability. Despite these benefits, our study demonstrates that providers are not sufficiently using S-ACP to document patient preferences, even in tertiary-care settings. 

Our central finding is that, over eight years, across all types of outpatient clinics at our institution, only 4.2% of patients demonstrated evidence of documented S-ACP. A temporal trend of increased S-ACP documentation was observed. Importantly, S-ACP documentation rate should not be construed as a proxy for ACP documentation rate, as it is estimated that the majority of ACP documentation (70–80%) occurs in the narrative free-text [11]. Utilizing definitions for S-ACP similar to ours, prior studies have also observed low rates for S-ACP, ranging from 13% to 43.2% in both ambulatory and emergency department settings [4, 5].

The findings from this study both highlight deficiencies in S-ACP documentation, and suggest multiple opportunities for quality improvement. One challenging aspect of ACP documentation is the non-standardized nature of documentation methods. In our analysis, six different methods of S-ACP were recorded, with the most common being problem list diagnoses and scanned copies of signed legal documents (advance directives, living wills or POLST forms). The documentation formats did not include free-text within clinic or progress notes in the EHR. Notably scanned documents are the only form of ACP documentation which contains legal signatures. In a similar population to ours (> 65 years of age), Wilson et al. found approximately half of patients within a large healthcare network had any form of ACP, and of these patients only 33% had a scanned document [12]. It is likely that many patients without S-ACP documentation may have had discussions pertaining to end-of-life care; however, the bulk of such conversational content is difficult to access in the EHR [6, 13]. Even if ACP were documented, the recorded information may be incomplete or inaccurate, a barrier to quality care which can be improved by S-ACP [4, 14].

Consistent outreach and technological innovations in the EHR could play critical roles in promoting and improving S-ACP documentation. One institution developed a hospital-wide, multi-pronged intervention to promote an ACP “Navigator” within the EHR, which served as a central digital repository for S-ACP documents [7]. This intervention resulted in increased S-ACP documentation rates by 5.3% in the first month of implementation and 1.3% monthly increase in rates thereafter compared to pre-intervention [7]. A similar effort to create an EHR Navigator in a pediatric hospital in Texas resulted in a rapid improvement in code status changes supported by appropriate documentation [14]. Other groups investigated the utility of short educational sessions between outpatient clinic visits, staff advocates for ACP discussion, or EHR reminders for providers, which significantly increased the rate of ACP discussion [15–17]. While S-ACP is a preferred form of documentation, in certain cases only narrative free-text documentation may be available. In these cases, harnessing natural language processing may be a useful adjunctive method to efficiently and accurately determine patient preferences [11].

SHC introduced the Serious Illness Care Program (SICP), developed by Ariadne Labs, as an institution-wide effort to facilitate ACP discussions between patients and clinicians [18]. Elements of serious illness discussions and ACP were incorporated into the SHC EHR as a shared documentation template [19]. Identifying seriously ill patients who would likely benefit from ACP through a process informed by artificial intelligence, SHC’s program reached and sustained target S-ACP completion rates of 10% at various points between July 2020 and January 2021 [19].

The importance of S-ACP is also apparent from a regulatory and health policy perspective. In 2016, CMS began reimbursing providers for holding ACP discussions with their patients [20]. The existence of auditable documentation, such as provided by S-ACP, will enhance the ability of care networks to report this quality metric to CMS and other payers. Moreover, healthcare systems can more easily track trends and improvements in S-ACP documentation compared to free-text documentation. Quality improvement research would also benefit from adoption of S-ACP, since manual chart review currently is both time-consuming and often inaccurate [11].

The results of our study should be interpreted in light of certain limitations. Given the de-identified nature of data extraction, we had limited access to patient-level covariates (such as race or language). Race, language, education, and other contextualizing patient-level variables may significantly confound or mediate the relationships we observed. We also lacked granular provider-level data (such as individual provider identification). As such, we could not provide detailed insight into provider-level factors (such as years in practice, provider type) which may impact S-ACP documentation [21].

## Conclusion

In conclusion, in this retrospective study, we demonstrate a low rate of S-ACP documentation across ambulatory clinics in a large healthcare system. These data suggest that there exists a gap between provider-patient discussion and EHR-based documentation toward the end of life. Beyond S-ACP and in other areas of need for structured documentation, the importance of standardized documentation within rich-yet-complex EHR systems will only increase with the digitalization of healthcare.

## Supplementary Information

**Additional file 1:** **Supplemental Table 1.** Definitions of S-ACP in Electronic Health Records. Codes for structured advance care planning (S-ACP) documentation. Abbreviations: current procedural terminology (CPT), Physician Orders for Life-Sustaining Treatment (POLST). **Supplemental Table 2.** S-ACP Documentation Rate by Provider Type. Structured advance care planning (S-ACP) completion rates by provider type (includes providers that logged at least 450 patient encounters) based on encounters.

## Data Availability

Data referenced in this study will be available and provided upon request by lead author, Adela Wu (adelawu@stanford.edu).

## Abbreviations

ACP: Advanced Care Planning
CMS: Centers for Medicare and Medicaid Services
DNR: Do Not Resuscitate
POLST: Physician Orders for Life Sustaining Treatment
S-ACP: Structured Advanced Care Planning
SHC: Stanford Health Care
SICP: Serious Illness Care Program
