# Supplementary material for: Low rates of structured advance care planning documentation in electronic health records: results of a single-center observational study
Source: BMC Palliat Care. 2022 Nov 22;21:203. doi: 10.1186/s12904-022-01099-9 (PMC9686086; doi:10.1186/s12904-022-01099-9)
Supplement: Supplementary file 1 — Additional file 1: Supplemental Table 1. Definitions of S-ACP in Electronic Health Records. Codes for structured advance care planning (S-ACP) documentation. Abbreviations: current procedural terminology (CPT), Physician Orders for Life-Sustaining Treatment (POLST). Supplemental Table 2. S-ACP Documentation Rate by Provider Type. Structured advance care planning (S-ACP) completion rates by provider type (includes providers that logged at least 450 patient encounters) based on encounters. [file 12904_2022_1099_MOESM1_ESM.docx]

| Supplemental Table 1: Definitions of S-ACP in Electronic Health Records | |
| --- | --- |
| Type of S-ACP Documentation | **Codes Denoting Type of Documentation** |
| ACP Smartform | Smartform ID 1126 |
| CPT code | 99497, 99498 |
| Problem List Diagnosis | 2183647, 1103560, 1103561, 1168546, 1131459, 199135, 199146, 1031608, 1764366, 199136, 1764365, 1259418,1119861,1259401,1259402,1119862,199145,1231592,1857184,1231700,1764571,1764592,1844723,1232176,1855240,1227800,1762251,1855250,1855243 |
| Encounter Diagnosis | 2183647, 1103560, 1103561, 1168546, 1131459, 199135, 199146, 1031608, 1764366, 199136,  1764365,1259418,1119861,1259401,1259402,1119862,199145,1231592,1857184,1231700,1764571,1764592,1844723,1232176,1855240,1227800,1762251,1855250,1855243 |
| Scanned Document | Including Physician Orders for Life-Sustaining Treatment (POLST) form, Advance Directive, Living Will |
| Surrogate Decision Maker | Documentation of Surrogate Decision Maker |

**Supplementary Table 1:** Codes for structured advance care planning (S-ACP) documentation. Abbreviations: current procedural terminology (CPT), Physician Orders for Life-Sustaining Treatment (POLST)

| Supplemental Table 2: S-ACP Documentation Rate by Provider Type | | |
| --- | --- | --- |
| Provider Type | **Total Number of Patient Encounters** | **S-ACP Completion Rate** |
| Anesthesiologist | 9,962 | 6.89% |
| Case Manager | 533 | 9.38% |
| Clinical Nurse Specialist | 2,143 | 19.51% |
| Fellow | 14,945 | 9.25% |
| Genetic Counselor | 576 | 5.03% |
| Nurse Practitioner | 81,731 | 9.77% |
| Occupational Therapist | 4,441 | 9.05% |
| Physical Therapist | 6,537 | 11.46% |
| Physician | 454,862 | 9.30% |
| Physician Assistant | 27,596 | 9.72% |
| Psychologist | 3,685 | 8.52% |
| Referring Physician | 27,319 | 8.18% |
| Resident | 6,183 | 11.52% |
| Resource Liaison | 43,283 | 7.97% |
| Social Worker | 474 | 28.90% |
| Speech Language Pathologist | 4,574 | 10.38% |

**Supplemental Table 2:** Structured advance care planning (S-ACP) completion rates by provider type (includes providers that logged at least 450 patient encounters) based on encounters.
